# Repositioning of the humeral tuberosities can be guided by pectoralis major insertion

**DOI:** 10.1007/s11751-014-0205-z

**Published:** 2014-12-19

**Authors:** Alec Cikes, Étienne Trudeau-Rivest, Fanny Canet, Jonah Hébert-Davies, Dominique M. Rouleau

**Affiliations:** 1 Hôpital du Sacré-Cœur de Montréal (HSCM), 5400 Gouin Ouest, Local C-2095, Montreal, QC H4J 1C5 Canada; 2 Synergie-Medical Center, Rue du Grand-Pré 2B, 1007 Lausanne, Switzerland; 3 University of Montreal, 2900 Boulevard Edouard-Montpetit, Montreal, QC H3T 1J4 Canada

**Keywords:** Shoulder, Pectoralis major, Bicipital groove, MRI, Tuberosity, Proximal humerus fracture

## Abstract

In complex proximal humerus fractures, positioning of the tuberosities can be a challenge. This study demonstrates the constant angle between the pectoralis major (PM) and the medial lip of the bicipital groove (BG) on the horizontal axial plane. This angle can be used to determine the rotation, as well as the positioning of the tuberosities, when planning a hemiarthroplasty or a reconstruction. Thirty-one shoulder MRIs were reviewed by three independent observers. The measurements were taken by superposing the axial cut of the proximal humerus, at the level of the distal bicipital groove, and the cut at the top of the PM insertion. By aligning the centers of rotation, we could determine the arcs of rotation between the insertion of the PM and the lips of the medial and lateral bicipital groove (MBG and LBG). Both angles were compared in terms of reliability, reproducibility, and precision. The mean PM–MBG angle was 3.7° [standard deviation (SD) 14.7°] and 27.4° (SD 14.4°) for the PM–LBG angle. We obtained good and very good intra-class correlation coefficient (ICC) results for inter- (0.675) and intra-observer (0.793) reliabilities on the medial angle, plus excellent results for the lateral angle (inter-observers 0.962 and intra-observer 0.895). This study demonstrates that the repositioning of humeral tuberosities can be guided by pectoralis major insertion. This will help achieve proper positioning of the metaphysis in relation to the diaphysis during surgery for complex proximal humerus fractures.

## Introduction

**Fig. 1:**
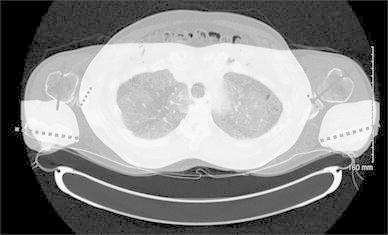
Example of malposition of a humeral subcapital fracture leading to later glenohumeral osteoarthritis

Proximal humerus anatomy varies substantially between individuals and even from side to side in the same individual [1]. Therefore, accurate knowledge of the osseous anatomy is very useful in the reconstruction of complex fractures [2]. Recent literature has identified standard values for tuberosity height in open reduction and internal fixation (ORIF) as well as implant height in shoulder arthroplasty [3–6]. While some recommend putting shoulder hemiarthroplasty in 20 degrees of retroversion for fractures [7], finding proper rotation in ORIF is challenging. While humerus rotation can be assessed reliably by computerized tomography (CT scan), as in this case of humeral malunion (Fig. 1), no intra-operative method is described.

Proximal humerus fractures are the third most frequent type of osteoporotic fractures [8], and surgical success depends on correct tuberosity positioning and healing [9]. Malposition of the tuberosities can lead to impingement and decreased range of motion [3, 9, 10]. As stated by Murray [11], establishing the rotation of metaphysis on the diaphysis is the last step of the reduction after controlling translation. There are two goals for reduction: The greater tuberosity height should be 5 mm lower than the top of the humeral head [12], and on the axial plane, both tuberosities should have even heights and be on either side of the bicipital groove [13].

Our study’s primary objective was to demonstrate the presence of a constant angle between the pectoralis major (PM) insertion on the humeral shaft and the bicipital groove (BG) in the axial plane. This angle could be used to determine the rotation and positioning of the tuberosities because even in Neer IV fractures, the fracture line between lesser and greater tuberosity rarely involves the bicipital grove [14, 15]. Two different angles will be compared: medial lip of the bicipital groove to PM insertion and lateral lip to PM insertion.

Our hypothesis is that the mean medial angle (PM–MBG) is less than 10 degrees, the lateral angle (PM–LBG) is greater than 10 degrees, and that these angles are reliable. The ideal landmark would present good inter- and intra-observer reliabilities, be the smallest possible, and show minimal variability.

## Materials and methods

The protocol was as follows: A database of randomly selected shoulder magnetic resonance imaging (MRIs) was reviewed by a musculoskeletal radiologist to confirm inclusion–exclusion criteria and to identify those that included the entire pectoralis major insertion that was needed for the study. All skeletally mature patients with all types of pathologies not affecting the anatomy of the humerus were included, but all MRIs performed for proximal humerus fractures, pathologies of the pectoralis major, or any other abnormality affecting bony anatomy (previous surgery, infection, tumor or dysplasia) were excluded. Thirty MRIs were deemed necessary, following the recommendations by Harrison and et al. [16], and because no established values exist for the measurements needed (from the distal most level of the insertion of the subscapularis at the distal bicipital groove and at the top of the PM insertion). There was no power study. Finally, all measurements were taken once, by three different observers, and they were then repeated in a separate setting by two of the three previously mentioned observers, blinded and in a different order, to evaluate intra-observer reliability.

### Measurement criteria

**Fig. 2:**
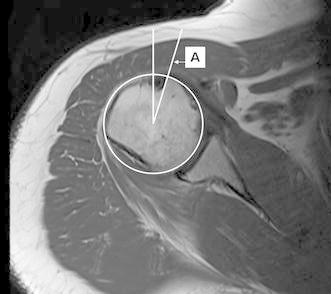
Axial cut of the proximal humerus at the level of the distal bicipital groove. *Line A* is drawn from the center of rotation to the medial lip of the bicipital groove at the level of the inferior insertion of the subscapularis muscle

**Fig. 3:**
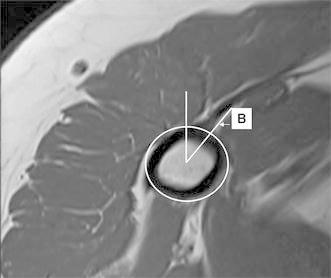
Axial cut of the proximal humerus at the level of the top of the PM insertion. *Line B* is drawn from the center of rotation to the medial aspect of the PM proximal insertion

**Fig. 4:**
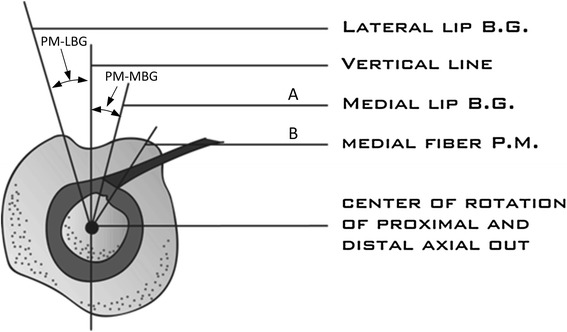
Schematic representation of the superposition of both axial cuts with PM–MBG and PM–LBG angles

 Measurement was taken by superimposing two axial cuts of the proximal humerus: one at the distal most level of the insertion of the subscapularis at the distal bicipital groove, and one at the top of the PM insertion. The center of rotation for each cut was defined as the center of a fitted circle applied on the axial cut. Then, two lines were drawn: the first (line A) from the center of rotation to the medial lip of the bicipital groove (Fig. 2), and the second (line B) from the center of rotation to the medial aspect of the PM proximal insertion (Fig. 3). The angle formed by these two lines was measured (Fig. 4). This angle was named “the rotation arc between the pectoralis major and medial bicipital groove” (PM–MBG). Another angle was measured in the same fashion using the lateral lip as the reference line and was named PM–LBG. Both angles were compared in terms of reliability, reproducibility, and precision.

All measurements were taken using a specialized validated software (SliceOMatic, Tomovision, Magog, QC, Canada).

### Statistics

Descriptive statistics were used to define the PM–MBG angle and PM–LBG angle. The Mann–Whitney test was used to compare mean PM–BG angles between males and females as our groups contained less than 20 subjects. Inter- and intra-observer reliabilities were tested using intra-class correlation coefficient (ICC) with the following standard classification: ICC 0.61–0.75 = good, ICC 0.76–0.9 = very good, and ICC > 0.91 = excellent.

## Results

A total of 31 MRIs were reviewed with a mean patient age of 54 years (25–86, SD 14).There were 15 males and 16 females, and the left shoulder was evaluated in 22 cases.

The mean PM–MBG angle was 3.7° (SD 14.7°), and the mean PM–LBG angle was 27.4° (SD 14.4°). The medial lip of the biceps gutter was the closest to the pectoralis major insertion.

There was no statistically significant difference in the PM–MBG angles between males and females (*p* > 0.3) for all angles tested with the Mann–Whitney test. There was good inter-observer correlation and reliability for the medial angle with a mean ICC of 0.675 (range 0.368–0.846), and it was excellent for the lateral angle with an ICC of 0.962 (range 0.926–0.982) (Table 1). We also obtained very good intra-observer correlation and reliability, with ICC values of 0.897 and 0.793 for observers one and two (PM–MBG).

**Table 1:** Results—inter-observers and intra-observer ICCs for PMMBG and PMLBG measurements

Reliability	Evaluators	Angle	ICC (95 % CI)
Inter-observer	Evaluators 1, 2, 3	PMMBG	0.675 (0.368–0.846)
		PMLBG	0.962 (0.926–0.982)
Intra-observer	Evaluator 1	PMMBG	0.897 (0.895–0.899)
		PMLBG	0.895 (0.890–0.898)
	Evaluator 2	PMMBG	0.793 (0.570–0.900)
		PMLBG	0.898 (0.799–0.949)

*ICC* intra-class correlation, *CI* confidence interval

## Discussion

**Fig. 5:**
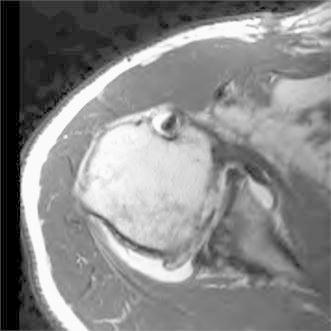
Secondary osteoarthritis in a case of severe malrotation

Ideally, proximal humerus fractures are treated by anatomic reduction using fracture lines as reference points. However, achieving this can be particularly complicated in four-part fractures with comminution. After restoration of the proximal humerus, it can be hard to assess and restore humerus rotation: too much retroversion will decrease internal rotation and affect functionality. Inversely, creating too much anteversion will decrease external rotation. Figures 1 and 5 are examples of cases of secondary arthritis with malunion in excessive retroversion. Malrotation can also cause problems in the fixation of the proximal humerus as the plate should be just lateral to the bicipital groove to be able to lie distally between the pectoralis major insertion and deltoid insertion.

Tuberosity positioning after surgery of a proximal humeral fracture is the key to a good clinical outcome [14]. Final tuberosity malposition is correlated with unsatisfactory results such as superior migration of the prosthesis, stiffness, weakness, and chronic pain [9]. Furthermore, tuberosity malposition and migration is linked with poor functional results because of the modified lever arm of glenohumeral abduction [17, 18].

The purpose of this study was to identify the presence of a reliable angle between the pectoralis major (PM) insertion on the humeral shaft and the bicipital groove (BG). Our results confirm that the medial aspect of the bicipital gutter is relatively parallel with the insertion of the pectoralis major proximal insertion. While it is a reliable measurement, the lateral edge should not be used to guide rotation because it is not in line with the tendon insertion. While there is a certain amount of variability, it is important to remember that the precision of angle measurements on MRI is much greater than on actual bone. Because an MRI allows more precise measurements, it thus gives a value of what the rotation should be and a fair estimate for the surgeon intra-operatively.

Therefore, we recognize that our study might not describe a perfect measurement method for MRI evaluation; however, we feel that this angle (PM–BMG) is useful intra-operatively for evaluating rotation. While a previous study exists evaluating rotator cuff insertions for tuberosity reduction, questions can be raised about its reproducibility [3]. Both the bicipital groove and the pectoralis major tendon are easily identifiable in open shoulder surgery and are thus ideal candidates for anatomic landmarks. Our measurements showed both good inter- and intra-observer reliabilities, demonstrating that this angle (PM–BMG) is reliable. This landmark is particularly useful in complex and comminutive fractures such as a fracture dislocation or a Neer IV fracture that are classically treated by open deltopectoral approach.

By using this anatomic marker, malposition could be avoided. Using these findings and others previously described [11], we can now devise an evidence-based protocol for the reduction in fragments in Neer IV complex proximal humeral fracture (three- and four-part fractures) in cases of open reduction and internal fixation (ORIF):

1. Exposing the humeral head by tuberosity mobilization and rotator interval opening.
2. Restoring the humeral head to 20 degrees [6] retroversion and 130 degrees of neck angle, with temporary fixation of the head to the glenoid with small smooth K-wires.
3. Reducing the tuberosities around the humeral head (the rotation will be determined by rotator cuff tensioning) [9].
4. The GT needs to be 5 mm lower than the humeral head [12].
5. The height of the humeral head is then adjusted 5.5 cm [19] from the pectoralis major proximal fiber with temporary wire and sutures.
6. Finally, the rotation of the proximal humerus in relation to the diaphysis is corrected to adjust the medial bicipital gutter to the medial insertion of pectoralis major fibers.
7. Definitive fixation of fragments is performed.

## Conclusion

Our study demonstrated that, in normal shoulders, the medial edge of the bicipital groove is relatively parallel to the insertion of the pectoralis major tendon. These two easily identifiable bony landmarks can then be used to evaluate rotation during osteosynthesis even in very severe fractures. Further studies are needed to determine whether this method is as applicable as we believe it to be in an operative setting. Nevertheless, it remains a useful adjunct for surgeons performing ORIF on complex proximal humerus fractures.
